# Supplementary figures and images for: Metalloproteomics Reveals Multi-Level Stress Response in Escherichia coli When Exposed to Arsenite
Source: Int J Mol Sci. 2024 Sep 2;25(17):9528. doi: 10.3390/ijms25179528 (PMC11394912; doi:10.3390/ijms25179528)

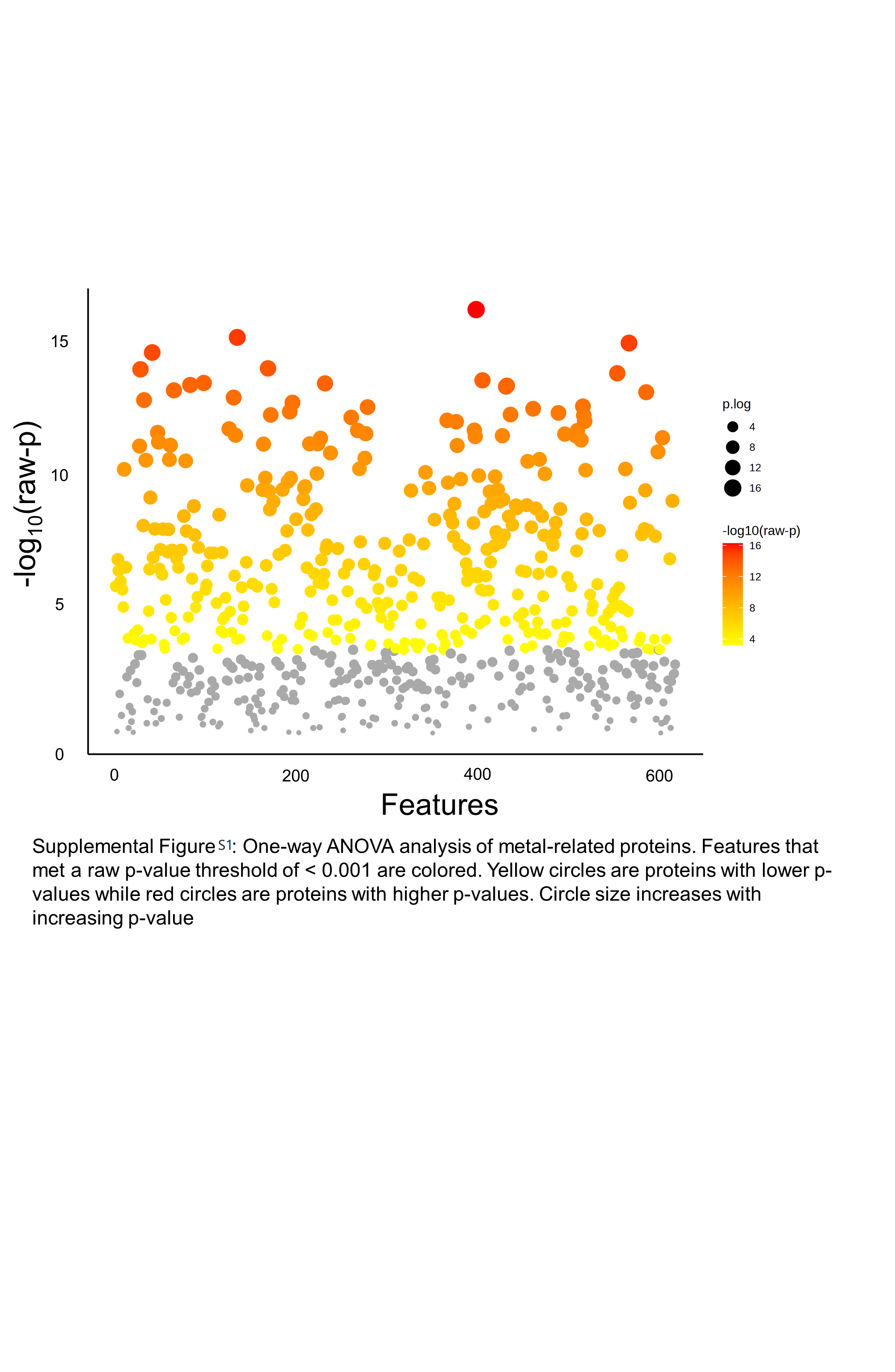

Supplement: Supplementary file 1 [file ijms-25-09528-s001.zip › SuppFig_S1.tif]

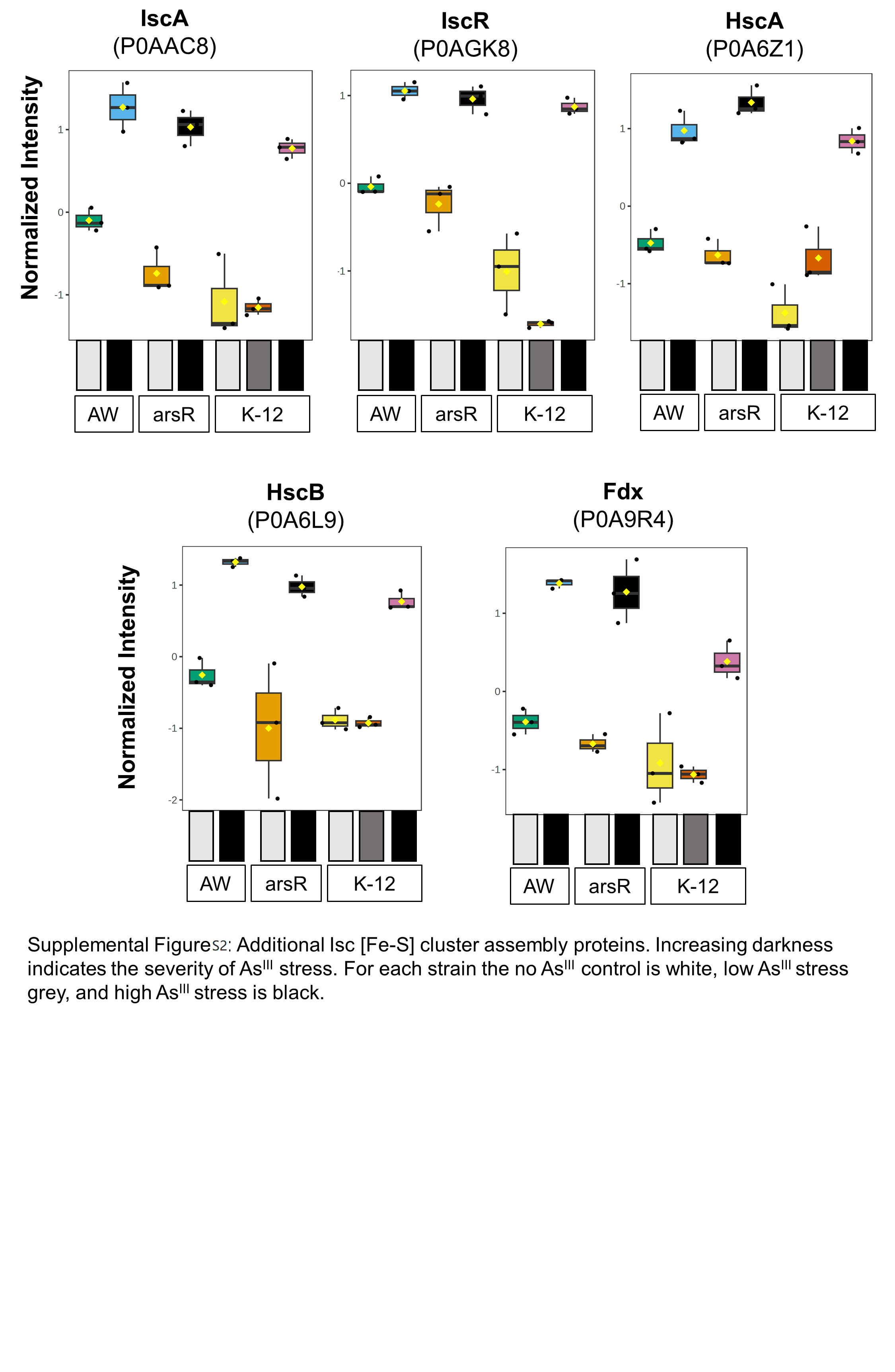

Supplement: Supplementary file 1 [file ijms-25-09528-s001.zip › SuppFig_S2.tif]

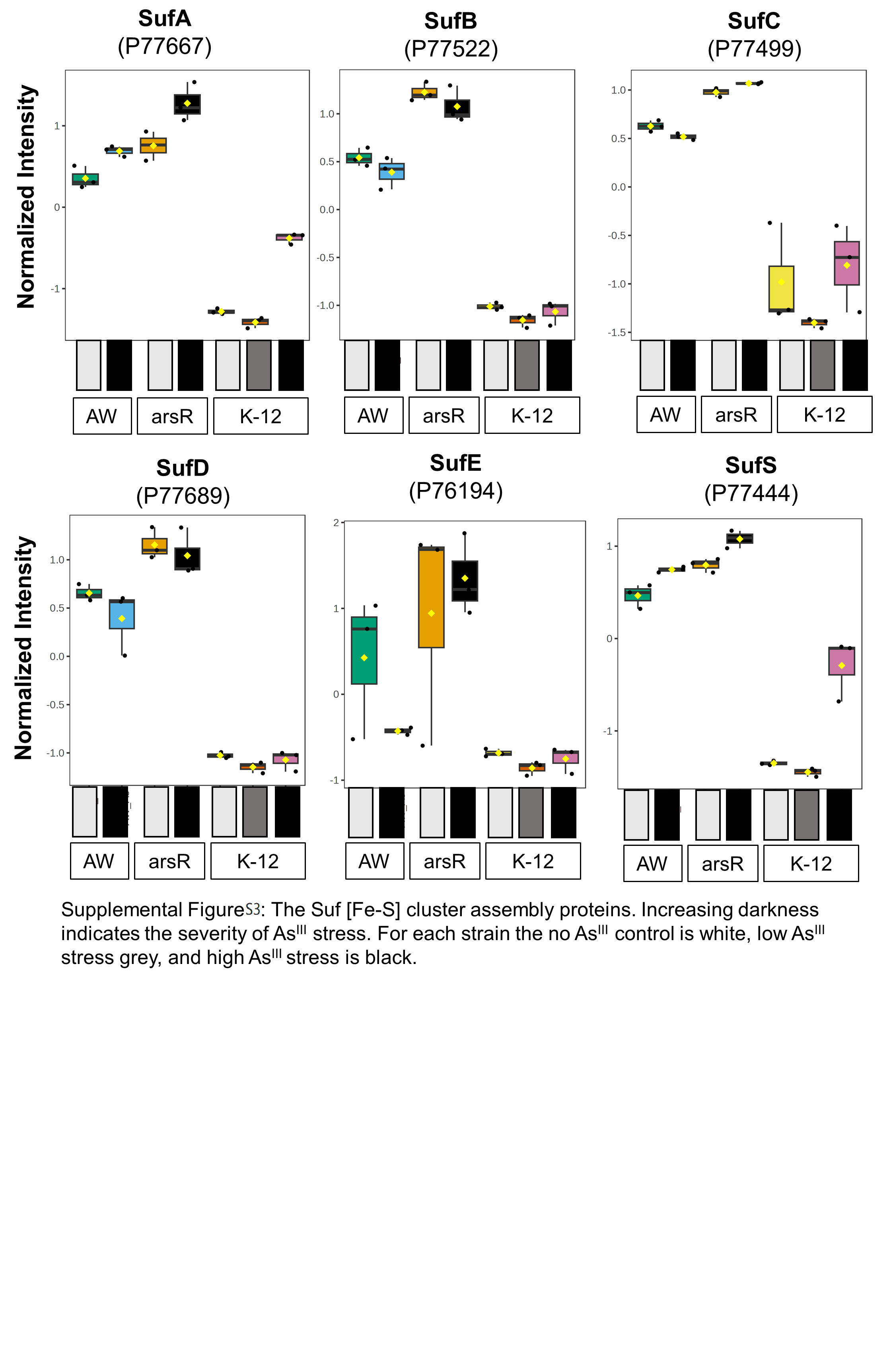

Supplement: Supplementary file 1 [file ijms-25-09528-s001.zip › SuppFig_S3.tif]

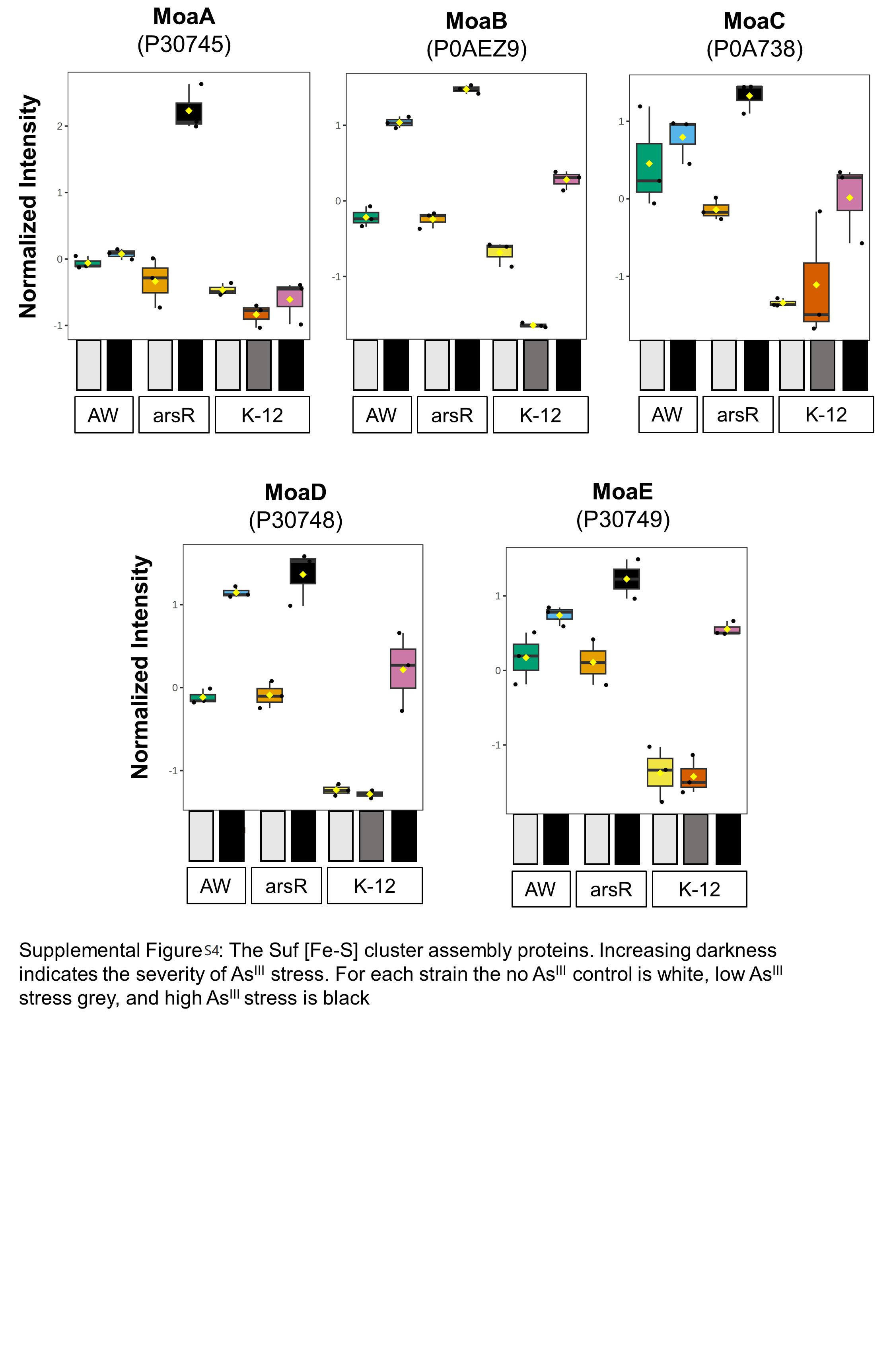

Supplement: Supplementary file 1 [file ijms-25-09528-s001.zip › SuppFig_S4.tif]

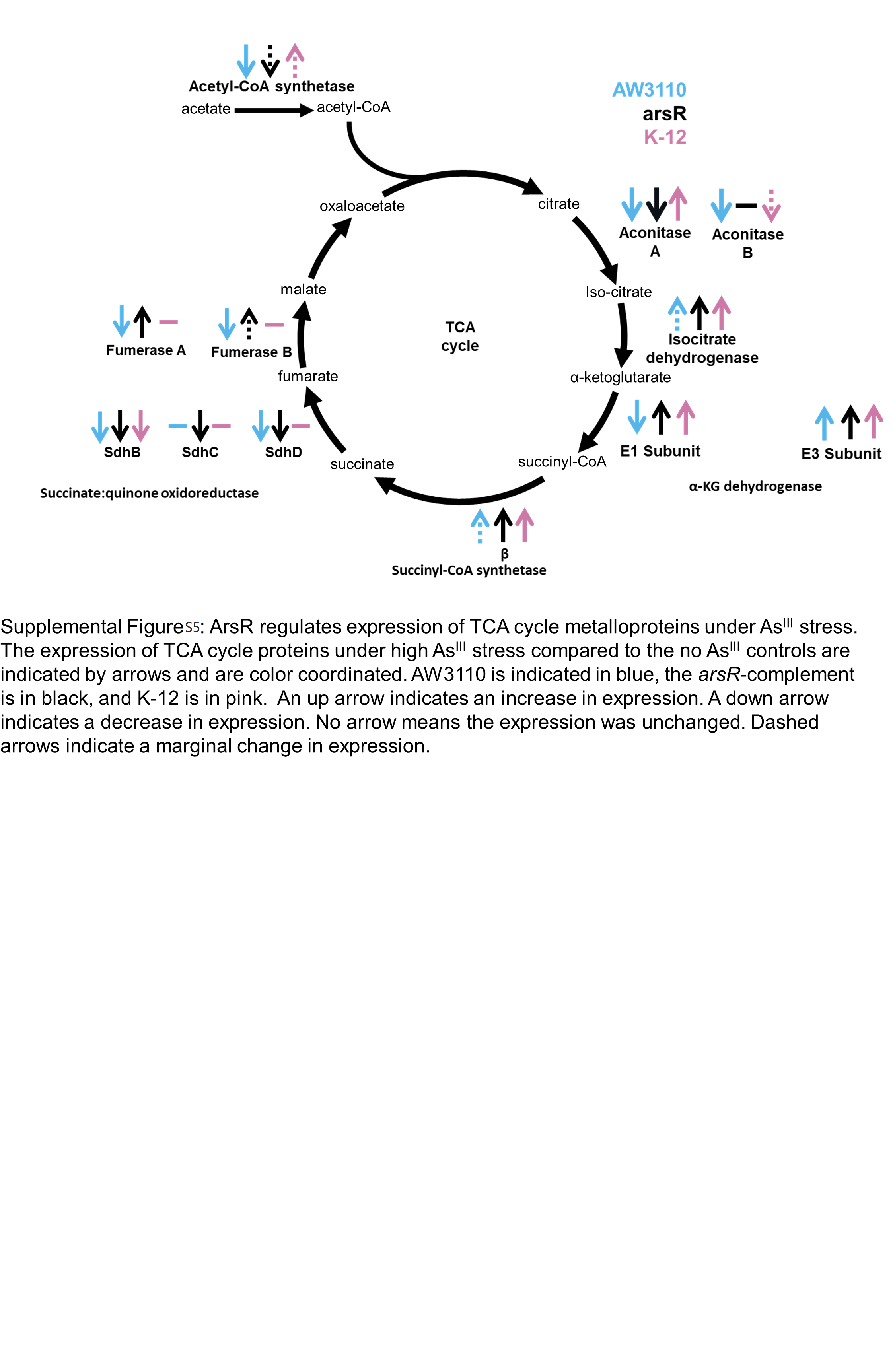

Supplement: Supplementary file 1 [file ijms-25-09528-s001.zip › SuppFig_S5.tif]
